# Genetic Testing in Periodontitis: A Narrative Review on Current Applications, Limitations, and Future Perspectives

**DOI:** 10.3390/genes16111308

**Published:** 2025-11-01

**Authors:** Clarissa Modafferi, Cristina Grippaudo, Andrea Corvaglia, Vittoria Cristi, Mariacristina Amato, Pietro Rigotti, Alessandro Polizzi, Gaetano Isola

**Affiliations:** 1 UOC Genetica Medica, Fondazione Policlinico Universitario A. Gemelli IRCCS, 00168 Rome, Italy; clarissamodafferi@libero.it; 2 Dipartimento Universitario Testa Collo ed Organi di Senso, Università Cattolica del Sacro Cuore, 00168 Rome, Italy; 3 UOC Clinica Odontoiatrica, Dipartimento di Neuroscienze, Organi di Senso e Torace, Fondazione Policlinico Universitario A. Gemelli IRCCS, 00168 Rome, Italy; 4 Department of General Surgery and Surgical-Medical Specialties, School of Dentistry, University of Catania, 95123 Catania, Italypietro.rigotti1999@libero.it (P.R.); gaetano.isola@unict.it (G.I.); 5 International Research Center on Periodontal and Systemic Health “PerioHealth”, University of Catania, 95124 Catania, Italy

**Keywords:** periodontitis, genetic testing, oral microbioma, personalized medicine, systemic diseases

## Abstract

**Background**: Periodontitis is a multifactorial inflammatory disease with a complex interplay between microbial, environmental, and host-related factors. Among host factors, genetic susceptibility plays a significant role in influencing both disease onset and progression. Over the past two decades, a wide range of genetic tests, ranging from single-nucleotide polymorphism (SNP) analysis to genome-wide association studies (GWAS), have been explored to assess individual risk profiles and potential treatment responses. However, despite initial enthusiasm, the clinical integration of genetic testing in periodontics remains limited. This narrative review aims to critically examine the current landscape of genetic testing in periodontitis, including commercially available tests, their scientific validity, and their clinical utility. **Methods**: Most relevant studies which were published in recent years were identified by using the major scientific search engines, including PubMed, Scopus, and Web of Science. Articles discussing genetic susceptibility, key gene polymorphisms, and emerging technologies were included in this narrative review. **Results**: Polymorphisms in genes coding for *IL-1*, *IL-6*, *TNF-α*, and in others involved in immune modulation and bone metabolism, are associated with periodontitis. Nevertheless, there are limitations related to heterogeneity in study design, population stratification, and gene–environment interactions. Moreover, emerging technologies, including polygenic risk scoring and machine learning approaches, may enhance the predictive value of genetic tools in periodontology. **Conclusions**: A deeper understanding of genetic susceptibility could pave the way for precision dentistry and personalized periodontal care, but significant hurdles remain before genetic testing can become a routine component of periodontal diagnostics.

## 1. Introduction

Periodontitis is a chronic multifactorial inflammatory condition associated with dysbiosis characterized by progressive destruction of the tooth-supporting structures, including periodontal ligament, gums, cementum, and alveolar bone [1]. It is caused by a complex interplay of microbial, environmental, and host-related factors. In fact, in genetically predisposed individuals, oral dysbiosis together with other environmental factors, such as smoking or other systemic conditions including diabetes, cardiovascular diseases, and neurological diseases, leads to aberrant inflammation, which damages periodontal tissues [2,3,4,5,6,7]. Host susceptibility plays a vital role in the onset and the progression of periodontitis. For this reason, during the last decades there has been an increasing interest in identifying genetic profile risk in order to facilitate diagnosis and to tailor treatment. Genetic tests, including single-nucleotide polymorphism (SNP) analysis and genome-wide association studies (GWAS) have been proposed. SNP analysis detects specific SNPs, which are alterations of coding or non-coding regions of the DNA [8]. When they affect coding regions, they can alter or non-alter the sequence of amino acids of a coded protein; meanwhile, when they are in non-coding regions, they can affect gene regulation, mRNA stability, or splicing [9]. On the other hand, GWAS are large-scale investigations that analyze the genome for common genetic variants, including SNPs, to identify associations with specific conditions or diseases without prior assumptions about candidate genes [10]. In GWAS, allele frequencies between affected individuals and healthy controls are compared [11]. Currently, SNP analysis and GWAS have detected genes polymorphisms associated with periodontitis in genes coding for pro-inflammatory cytokines, including *IL-1*, *IL-6*, *TNF-α*, and others involved in immune modulation and bone metabolism [12,13]. These results explain why dysbiosis alone is not sufficient to develop periodontitis; in fact, in predisposed people with the aforementioned genetic alterations, dysbiosis leads to severe host-inflammatory response which damages the periodontal structures. These findings about the genetic profiles of periodontitis patients are promising, but the application of genetic tests in ordinary clinical practice is still far away. In fact, the current studies about the genetic tests of periodontitis lack homogenization. Additionally, genetic profile is not sufficient, and the gene–environment interactions must be considered too. For this reason, this narrative review aims to provide a panoramic view of current genetic testing in periodontitis, including commercially available tests, their scientific validity, their clinical utility, and their limitations. Emerging technologies, including polygenic risk scoring and machine learning approaches are also discussed in order to highlight their potential roles in diagnosis and in tailoring periodontitis therapy.

## 2. Materials and Methods

For this narrative review, a comprehensive literature search was conducted using two major electronic databases: PubMed and Google Scholar. The search included studies published between January 2000 and June 2025, and the following keywords were used in combination through Boolean operators: “Periodontitis”, “Genetic testing”, “Host susceptibility”, “IL-1 polymorphism”, and “Precision dentistry”. Initially, duplicate records and articles not pertinent to the topic or not meeting the inclusion criteria were excluded. Titles and abstracts were screened independently by two researchers, and full texts were retrieved for those considered potentially eligible. Disagreements regarding inclusion were resolved through discussion with a third reviewer, and final decisions were made by consensus. Only studies written in English were considered. Randomized controlled trials, cohort studies, case–control studies, case series, systematic reviews, and meta-analyses were included in the selection. Gray literature, such as conference abstracts, theses, or preprints not yet peer-reviewed, was excluded. To ensure scientific rigor, all selected studies underwent quality assessment using appropriate tools depending on the study design: the Cochrane Risk of Bias Tool 2.0 for randomized trials, the Newcastle–Ottawa Scale 2023 for observational studies, and AMSTAR 2 for systematic reviews and meta-analyses. Only studies meeting a minimum methodological quality threshold were included in the final synthesis.

## 3. Genetic Architecture of Periodontitis

### 3.1. Inflammatory Cytokine Genes in Periodontitis

Genetic susceptibility to periodontitis has been extensively investigated through a variety of approaches, including candidate gene studies and genome-wide association studies (GWAS). Early investigations focused on polymorphisms in genes encoding pro-inflammatory cytokines such as *IL1A*, *IL1B*, *IL6*, and *TNFA*, given their central roles in immune response and inflammation [14]. The *TNF-α* promoter polymorphism at −308 (G>A, rs1800629) has been extensively investigated in periodontitis. While several individual case–control studies reported associations with disease susceptibility, pooled evidence from systematic reviews and meta-analyses indicates only small effect sizes and substantial between-study heterogeneity, with some ancestry-specific signals reported (stronger effects in some Asian cohorts). After accounting for study quality and potential publication bias, meta-analyses conclude that *TNF-α* −308 genotyping alone has limited clinical utility as a predictive marker for common forms of periodontitis. Therefore, *TNF-α* remains of research interest but is not presently a robust standalone biomarker for routine clinical risk stratification [15,16,17,18,19]. Among these, the composite *IL1* genotype, especially the combination of *IL1A* −889 C>T and *IL1B* +3954 C>T polymorphisms, has been most consistently linked to increased periodontal disease severity, particularly in European populations [15]. However, replication across diverse ethnicities has yielded mixed results, pointing to population-specific genetic architectures and the influence of environmental modifiers [16].

While *IL1A*, *IL1B*, *IL6*, and TNFA are among the most extensively studied cytokine genes in periodontitis, numerous other pro- and anti-inflammatory mediators have been implicated in disease susceptibility, progression, and tissue destruction. Genetic polymorphisms affecting these mediators can influence the magnitude and duration of the host-immune response to periodontal pathogens. Several candidate gene studies have investigated *IL-1* polymorphisms (notably *IL1A* −889 and *IL1B* +3954/rs1143634) in relation to periodontitis. While individual studies have reported positive associations, pooled evidence from systematic reviews and meta-analyses indicates modest and inconsistent effects, with results that vary by disease phenotype and ancestry. For instance, some meta-analyses report associations in European-ancestry cohorts, whereas others, including analyses focused on aggressive periodontitis, find no clear link, highlighting limited generalizability and modest predictive utility of IL-1 genotyping for routine clinical risk stratification [20,21].

For example, *IL10*, a key anti-inflammatory cytokine, downregulates the expression of *IL1* and *TNFA*. Polymorphisms such as *IL10* −1082 A>G have been associated with altered IL10 expression and an increased risk of chronic periodontitis [17]. On the other hand, pro-inflammatory cytokines of the Th17 pathway, including *IL17A* and *IL17F*, play crucial roles in driving neutrophil recruitment, matrix metalloproteinase (MMP) production, and osteoclast activation via RANKL signaling [18].

*IL8* (CXCL8) acts as a potent chemoattractant for neutrophils and is significantly upregulated in periodontal lesions. Polymorphisms in the *IL8* promoter may influence the host’s acute-phase response and susceptibility [22]. Moreover, osteoimmune regulators such as RANKL and OPG modulate the balance between bone resorption and preservation; their expression levels are directly affected by inflammatory cytokines [20,21].

Additional genetic contributors include matrix metalloproteinases (*MMP1*, *MMP8*, *MMP9*), which are key enzymes in the degradation of collagen and extracellular matrix in periodontal tissues, and Toll-like receptors (TLR2, TLR4), which mediate pathogen recognition and initiation of the innate immune response. Polymorphic variants in these receptors may alter signaling thresholds, thereby affecting the severity of periodontal inflammation [22].

Together, these genes define a complex immunogenetic landscape, in which the interplay of multiple variants—each with modest individual effects—may collectively determine periodontal disease risk and progression. We summarize the key cytokines implicated in periodontitis in Table 1.

### 3.2. GWAS-Identified Susceptibility Loci for Periodontitis

Subsequent GWAS have identified several loci associated with susceptibility to periodontitis. Among the most consistently reported are variants near *SIGLEC5*, *DEFA1A3*, and *GLT6D1*, which are functionally linked to key components of the innate immune system and mucosal integrity.

*SIGLEC5* encodes a member of the sialic acid-binding immunoglobulin-like lectins (Siglecs), specifically expressed on myeloid cells, including neutrophils and monocytes. It plays an immunomodulatory role by dampening inflammatory responses through the recruitment of SHP-1 phosphatase following ligand binding [34]. Pathogenic variants in *SIGLEC5* have been associated with altered neutrophil activation and impaired clearance of periodontal pathogens, contributing to a dysregulated inflammatory response [34].

*DEFA1A3* encodes α-defensins 1–3 (also known as human neutrophil peptides 1–3, or HNP1–3), small antimicrobial peptides stored in the azurophilic granules of neutrophils. These molecules exhibit broad-spectrum antimicrobial activity and are involved in direct microbial killing as well as chemoattraction of immune cells [35]. Copy number variation (CNV) in the *DEFA1A3* cluster has been linked to altered salivary defensin concentrations and differential susceptibility to chronic periodontitis [36].

*GLT6D1* encodes a putative glycosyltransferase, although its exact substrate specificity and biological role remain incompletely characterized. GWAS have implicated *GLT6D1* pathogenic variants, particularly rs1537415 on chromosome 9q34.3, in chronic and aggressive periodontitis [37]. Functional studies suggest that the risk allele reduces binding of the transcription factor GATA 3 in T cells. Although these associations provide biologically plausible links between genetic variation and periodontal pathogenesis, many findings have yet to be independently validated in large, multi-ethnic cohorts. Furthermore, the modest effect sizes and context-dependent penetrance underscore the need for integrative models that incorporate both genetic and environmental variables [38,39]. Genetic confirmation of *CTSC* mutations can aid in family counseling, guide screening of at-risk siblings, and alert clinicians to possible systemic associations such as skin disorders or immune dysfunction, thereby influencing both dental and medical management strategies.

Beyond these canonical loci, more recent GWAS and population-based studies have identified several additional candidate genes:

In an isolated Italian population, a significant association was found with *EFCAB4B*, a gene involved in calcium signaling. Though the mechanism remains unclear, the variant may influence immune-cell activation or epithelial responses [40].

A Finnish GWAS reported suggestive associations near *LAMA2* (encoding the laminin α2 subunit, involved in extracellular matrix stability), *ARHGAP18* (a regulator of cytoskeletal dynamics), and *HAS2/HAS2-AS1* (enzymes and antisense RNAs regulating hyaluronic acid synthesis) [41].

A large GWAS using UK Biobank data identified new loci including RP11-61G19.1, a long non-coding RNA, and HIST1H3L, encoding a histone protein involved in chromatin architecture. Although statistically significant, these findings await functional validation and replication [42].

Importantly, whole-exome sequencing (WES) studies have started to explore the role of rare coding variants in periodontitis. Although GWAS typically identify common variants with small effect sizes, WES allows for the detection of rare, potentially deleterious mutations in protein-coding regions. Preliminary WES-based analyses have implicated rare variants in immune regulation and epithelial integrity pathways, although these findings are still emerging and often limited by cohort size and phenotypic heterogeneity. Collectively, these findings underscore the complexity of the genetic landscape in periodontitis, involving both common and rare variants, protein-coding and regulatory elements, and a wide array of biological processes. While current genetic discoveries have yet to translate into routine clinical tools, they provide a foundation for developing polygenic risk models and understanding gene–environment interactions in the pathogenesis of periodontal disease. We summarize the main genetic loci implicated in periodontitis in Table 2.

### 3.3. Polygenic Risk Scores in Periodontitis

Polygenic risk scores (PRS) have emerged as a promising approach to capturing the cumulative effect of multiple common genetic variants associated with periodontitis, each of which may have only a small individual impact. Unlike traditional candidate gene approaches, PRS incorporate genome-wide SNP data to estimate an individual’s genetic liability to disease [45]. This method allows for stratification of patients into different risk categories, potentially guiding preventive and therapeutic strategies in precision periodontology [46]. Preliminary studies have shown that individuals with higher PRS for chronic periodontitis have significantly greater clinical attachment loss and alveolar bone resorption compared to those with low scores [47]. Moreover, PRS has shown additive predictive value when combined with conventional risk factors such as smoking and diabetes, suggesting its utility in multifactorial risk models [48]. However, the majority of PRS models are currently derived from European ancestry datasets, limiting their applicability in other populations due to differences in allele frequencies and linkage disequilibrium structures [36].

Another important limitation is the lack of consensus regarding which SNPs to include in PRS construction for periodontitis. Studies vary widely in their SNP selection criteria, statistical methods, and thresholds, making cross-comparisons difficult and hindering clinical translation [49]. Furthermore, although PRS provides probabilistic estimates of risk, it does not account for gene–environment interactions or epigenetic modulation, both of which are critical in the pathogenesis of periodontal disease [50]. Despite these challenges, ongoing efforts to refine PRS methodologies—such as ancestry-specific risk modeling, inclusion of rare variants, and integration with machine learning classifiers—may enhance their predictive performance and clinical relevance in the near future [51].

## 4. Host Susceptibility to Periodontal Disease: Emerging Molecular and Genetic Indicators

In recent years, a growing body of research has focused on identifying molecular characteristics that may predispose individuals to periodontitis. This includes the study of gene variants, epigenetic modifications, protein-based indicators, and transcriptomic alterations. These molecular signatures are increasingly regarded not only as tools for understanding disease mechanisms but also as potential aids in classifying risk profiles and tailoring personalized treatment or preventive protocols.

Among the molecules released during periodontal inflammation, several host-derived mediators have emerged as potential biomarkers. Notably, the enzymatically active form of matrix metalloproteinase-8 (aMMP-8), deeply involved in collagen breakdown and tissue destruction, has shown strong diagnostic potential. Hopealaakso et al. [37] demonstrated that elevated aMMP-8 concentrations—quantified via a non-invasive oral rinse assay (PerioSafe^®^ Sweden & Martina Due Carrare (PD)-Italy/ORALyzer^®^ Dentognostics Solingen, Germany)—were significantly linked with both systemic conditions such as metabolic syndrome (*p* = 0.005) and diabetes (*p* = 0.03), as well as with signs of ongoing periodontal inflammation. This rapid chairside test provides real-time insight into connective tissue degradation, even in patients who do not present with overt clinical symptoms, making it one of the few molecular diagnostics currently available for routine use in dental practice.

Gingival crevicular fluid (GCF) represents another valuable diagnostic matrix, offering a localized snapshot of periodontal inflammatory activity. Sedghi et al. (2021) [52] advocated for the use of multiplex assays targeting GCF biomarkers—such as *IL-1β*, *IL-6*, *TNF-α*, and various neutrophil-associated enzymes—to assess disease severity and predict progression. Although their contribution was largely theoretical, it referenced empirical studies that consistently found elevated IL-1β and aMMP-8 levels in sites of active tissue damage. Bibi et al. (2021) [53] expanded on this concept by reviewing numerous investigations in which increased levels of calprotectin and *IL-6* in GCF were strongly correlated with periodontal clinical parameters, including attachment loss and bleeding on probing (typically with *p* < 0.01). Despite the promising specificity and sensitivity of these analytes, the review also highlighted technical challenges, including inconsistencies in sample acquisition and variability among detection technologies, which currently limit clinical implementation.

From a genetic perspective, several polymorphisms have been explored for their association with periodontal susceptibility. Among the most reproducible findings are the *IL-1A* (−889 C/T) and *IL-1B* (+3954 C/T) variants. A meta-analysis by Dommisch et al. [38] confirmed these polymorphisms as statistically significant risk factors, with pooled odds ratios of 1.35 (95% CI: 1.17–1.56; *p* < 0.001) and 1.34 (95% CI: 1.18–1.52; *p* < 0.001), respectively. These results align with previous studies, including those by López et al. [39] in diabetic populations, and by Dahash and Hussein [40] as well as Lohinai et al. (2023) [54], who described additional IL-1-related variants with either protective or risk-enhancing effects.

In contrast, other SNPs have not demonstrated consistent associations with disease. For example, investigations into *MMP-13* (rs2252070) and *MMP-8* (−799 C/T), discussed by Chatterjee and Rajasekar [41] and Dommisch et al. [38], revealed no significant or reproducible links with periodontitis. Similarly, polymorphisms in *ICAM-1* (rs5498), *SRXN1* (rs6053666), and *TLR4* (Asp299Gly) yielded negative or equivocal findings, often complicated by population heterogeneity or methodological variability.

Despite extensive research, no genetic test for periodontal risk stratification has received clinical regulatory approval. Current applications remain confined to investigational settings, with existing SNP panels requiring further validation. These findings underscore the limited predictive capacity of isolated genetic markers and the importance of contextualizing genetic data within broader biological and clinical frameworks. Moreover, just a limited number of genes have been clearly associated with periodontitis. For this reason, genetic tests in dentistry are not yet widely used and validated. Extending WES to patients with a well-founded clinical suspicion would be valuable, as it could enable the identification of additional causative genes; in this way, genetic tests would be an important and reliable tool for diagnosing periodontitis. To date, the only molecular diagnostic tool approved for routine use remains the aMMP-8 rapid assay, which has shown clear utility in detecting active periodontal breakdown and correlations with systemic inflammatory conditions. Among genetic candidates, the IL-1 gene cluster remains the most robust, though practical implementation is still limited. Meanwhile, GCF-based biomarker panels and transcriptomic signatures offer future promise but face obstacles related to standardization and reproducibility.

In conclusion, molecular profiling holds substantial potential to revolutionize the management of periodontal disease, shifting toward more personalized and predictive models of care. However, the translation of these insights into everyday clinical use remains a work in progress, with further validation and technological refinement required.

## 5. The Role of the Oral Microbiome in Periodontal Disease

The pathogenesis of periodontitis is closely tied to disruptions in the oral microbiome—an ecological community of microorganisms that, in health, exists in a relatively stable, symbiotic balance. A consistent finding across studies is that periodontitis is not the result of a single infection, but rather a consequence of microbial dysbiosis, in which pathogenic species expand at the expense of commensal bacteria.

High-throughput sequencing studies, particularly 16S rRNA gene sequencing of the V3–V4 regions, have shown that patients with periodontitis harbor a more diverse and ecologically complex microbiome, enriched in Gram-negative anaerobes such as *Porphyromonas gingivalis* (*P. gingivalis*), *Treponema denticola*, *Tannerella forsythia*, *Fusobacterium nucleatum*, *Filifactor alocis*, and *Prevotella intermedia* [42,55]. These organisms co-aggregate and engage in syntrophic relationships that enhance their virulence and persistence.

Distinct microbial profiles are observed between chronic and aggressive forms of periodontitis, with *Aggregatibacter actinomycetemcomitans* and *F. alocis* more abundant in the latter [56]. Additionally, the microbial composition of peri-implant pockets often mirrors that of neighboring teeth, as shown by Aoki et al. [46], with significant inter-site transmission of *P. gingivalis*, *T. denticola*, and *A. actinomycetemcomitans* (ORs 4.8–7.5; *p* < 0.02).

The diagnostic evaluation of the oral microbiome involves the analysis of microbial DNA from various sample types. Subgingival plaque, collected with sterile curettes, remains the gold standard for local microbiome assessment, offering high specificity for detecting pathogens. However, it is invasive and technique sensitive. In contrast, unstimulated saliva is a non-invasive and highly reproducible sample that reflects global microbial and inflammatory statuses. Studies by Kageyama et al. [57] and Jung et al. [58] demonstrated a strong correlation between salivary and subgingival microbiota (R = 0.86; *p* < 0.001), supporting the clinical relevance of salivary profiling.

Saliva also enables simultaneous quantification of host-derived biomarkers, including inflammatory cytokines (e.g., IL-8) and small extracellular vesicle (sEV)-associated miRNAs such as miR-146a and miR-155, which are elevated in periodontitis and modulate immune pathways [59,60]. IL-8 levels in saliva were significantly higher in patients with active periodontitis (*p* < 0.001), and correlated with the abundance of *Treponema*, *Prevotella*, and *Fusobacterium*.

Other sampling sites such as oral mucosa are useful in edentulous patients or for comparison between sites, although they tend to yield less specific profiles due to a broader commensal flora. Gingival crevicular fluid (GCF) is widely used for cytokine and enzymatic assays (e.g., IL-1β, aMMP-8) but is less suitable for microbiome analysis due to its small volume and contamination risk.

Currently, no microbiome-based diagnostic test has been commercialized for periodontal disease.

Emerging technologies like shotgun metagenomics and metatranscriptomics allow for functional profiling of bacterial communities and metabolic activity, identifying gene expression changes and microbial pathways involved in inflammation and tissue degradation. These techniques, however, remain restricted to research due to high costs and technical complexity.

Together, these findings support a new diagnostic paradigm: periodontitis arises from a shift in microbial ecology and host–microbiome communication, and both taxonomic and functional markers should be evaluated. Salivary microbiome profiling, inflammatory cytokine measurement (e.g., IL-8), and vesicle-contained miRNAs represent promising, though not yet fully validated, tools for personalized and non-invasive risk stratification in periodontology.

## 6. Genetic Polymorphism in Gingivitis

Periodontitis is part of a larger number of diseases called periodontal diseases, which affect periodontal structures but in different ways. Of these, there is gingivitis, which is a reversible inflammatory condition of the gums caused by dental biofilm accumulation [47,48,61] and characterized by the following signs: gingival bleeding, gingival redness, edema, and the absence of periodontal attachment loss [49,50]. Gingivitis can also be caused by other conditions; in fact, there is a distinction between plaque-induced gingivitis and non-plaque-induced gingivitis, which refer to a wide range of gingivitis, including necrotizing, plasma cell, viral, fungal, or bacterial gingivitis [51]. Gingivitis. if left untreated and depending on the host’s susceptibility, can evolve into periodontitis [62]. For this reason, some studies have investigated the genetic background of gingivitis. Gingivitis is frequently detected in young individuals. Some studies have investigated the association of genetic polymorphisms with gingivitis and caries. In fact, they are both non-communicable diseases that most frequently affect children. The VDR gene codes for the vitamin D receptor, containing many polymorphic regions [52]. It is a nuclear transcription factor that binds sites in the DNA, regulating a lot of genes and mediating the activity of vitamin D [53,63]. This last one is very important for the mineral composition of the teeth, but also for the immune response to bacterial insult, which explains its link with plaque-induced gingivitis [54,64,65,66,67,68]. Associations between vitamin D receptor (VDR) polymorphisms (FokI, BsmI, TaqI, ApaI) and periodontitis have been extensively studied, but results are heterogeneous. Early pooled analyses and subsequent studies report variable findings. For instance, Deng et al. [69] found ancestry-specific signals in Asians (ApaI AA associated with chronic periodontitis; weaker signals for BsmI/TaqI) while FokI showed no association; no associations were detected for aggressive periodontitis. More recent meta-analyses [56,70] confirm inconsistent results across phenotypes and populations, underscoring limited generalizability [69]. Barbosa et al. [57] analyzed the correlation between the genetic polymorphisms FokI (rs2228570) and BglI (rs739837) in VDR with dental caries and gingivitis susceptibility and did not find any correlation. Izakovicova et al. [58] investigated the VDR TaqI (rs731236) gene polymorphism with caries, and did not find any association; in contrast, they found a statistically significant (*p* < 0.05) association of such gene polymorphism with gingivitis, suggesting that it is implicated with its development. Thus, it is necessary to identify specific polymorphisms related to the disease, in this case TaqI (rs731236) gene polymorphism is correlated with the onset of gingivitis, but genetic polymorphisms FokI (rs2228570) and BglI (rs739837) in VDR are not.

Bartososova et al. [59] studied the allele and genotype frequencies of the *CD14* −260C/T polymorphism in children with and without plaque-induced gingivitis and the correlation with their plaque microbiota. They studied the polymorphism of CD14 because it has a role in the host-immune response to pathogens infecting gums. They did not find significant variations in the *CD14* −260C/T allele and genotype distribution among people affected and not affected by gingivitis; but it is interesting to highlight that children affected by gingivitis and with *P. gingivalis* carried the CT and TT genotypes more frequently than children affected by gingivitis and without *P. gingivalis* or healthy controls (*p* < 0.05). Such results suggest that the *CD14* −260C/T polymorphism works together with *P. gingivalis* to initiate the onset of plaque-induced gingivitis in the children who took part in the study.

Moreover, some genetic polymorphisms are not only related to the development of gingivitis, in fact, some scientists have also detected genetic polymorphisms which represent protective factors against gingivitis in children. Indeed, Dashash et al. [60] investigated the impact of the polymorphism of a variable number of tandem repeats of interleukin-1 receptor antagonist gene (*IL-1RN*) in Caucasian children affected by gingivitis. They detected a significant correlation between IL-1Ra gene polymorphism and gingivitis in children (*p* = 0.008), and, on the other hand, they demonstrated that the IL-1RN*2 allele (A2) was significantly more recurrent in controls than in gingivitis children (60% vs. 40% in children with gingivitis, *p* = 0.008), suggesting that it is protective against gingivitis.

Regarding the studies conducted in adults, Garlet et al. [71] detected that hyporesponsive SNPs, including *IL10*-592-C and *TLR4*-299-G, were prevalent in individuals affected by chronic gingivitis.

In another study [72], the gene polymorphism of TNF-α was identified as protective factor against gingivitis. This study analyzed the correlation between *TNF-*α (−308 G/A) polymorphism and gingivitis, and serum- and salivary-TNF-α concentrations, in a cohort of Mexican individuals. There were 171 subjects enrolled for this study, and they were distributed into two groups: the group of healthy subjects and the group of patients affected by gingivitis. PCR–RFLP assay was performed to analyze *TNF-*α (−308G/A) gene polymorphism. Samples of saliva and serum were taken, and cytokine level measurements were conducted through the ELISA analysis. The study demonstrated that *TNF-*α (−308G/A) polymorphism is protective in individuals who carry the A/A genotype and allele A. The G/A genotype correlates with augmented concentrations of high-density lipoprotein cholesterol (HDL-C) in the group of gingivitis patients. In healthy controls, increased concentrations of salivary-*TNF*-α and -*HDL*-C, and higher flow of saliva were registered; meanwhile, triglycerides, low-density lipoprotein cholesterol, and very low-density lipoprotein cholesterol concentrations were augmented in the group of gingivitis patients. Thus, it was proven that the *TNF-*α −308A/A genotype is protective against gingivitis.

In conclusion, thanks to the existing studies, there are some polymorphisms that can predict host susceptibility towards gingivitis, even in children (Table 3), but there are few studies about it, with different study designs, sample sizes, and methods.

## 7. Genetic Polymorphisms in Periodontitis and Gingivitis

As already discussed, gingivitis can evolve into periodontitis, even if not all cases of gingivitis do. Genetic polymorphisms can be involved in the onset of both conditions, but in different ways, and detecting gingivitis polymorphisms and periodontitis polymorphisms can be helpful to differentiate individual susceptibility towards the two conditions. Considering the inflammatory nature of both conditions, polymorphisms of genes coding for pro-inflammatory proteins and anti-inflammatory mediators have been detected in both gingivitis and periodontitis. In particular, according to the reported studies, genetic polymorphisms of *VDR* TaqI (rs731236), *CD14* −260C/T, *IL10*-592-C, IL-1Ra, and *TLR4*-299-G were associated with higher risk of developing gingivitis [58,59,60,71]. Meanwhile, the IL-1RN*2 allele (A2) and *TNF-*α (−308G/A) polymorphisms were suggested to be protective against gingivitis [60,72]. An increased risk of developing periodontitis is associated with genetic polymorphisms of *IL1A*, *IL1B*, *IL6*, and *TNFA* genes [14,38] and *MMP-9* (−1562C/T) [73], *ANRIL* rs1333048, rs1333042, rs2891168, and rs496892 polymorphisms might have impact on susceptibility to periodontitis, especially in Caucasian individuals [74]. Additionally, a weak relationship between *GSTT1* and *GSTP1* rs1138272 polymorphisms and periodontitis in non-smokers was observed [75]. To better evaluate the genetic profiles suggesting higher risk of periodontitis, Garlet et al. [71] proposed a different type of study, using chronic gingivitis as the reference condition in genetic studies of periodontitis. In this way, this approach would allow giving a clearer definition of susceptibility and resistance phenotypes, increasing the statistical power to detect genetic associations with the disease. They considered that in periodontitis research, the exposure concept is complicated because oral hygiene removes the microbial trigger, making it difficult to distinguish the truly resistant from the unexposed individuals. As a result, control groups are heterogeneous, with genotypic traits between periodontitis and gingivitis, which can bias case–control study outcomes. For this reason, in periodontitis genetic studies, the control group should be composed of chronic gingivitis patients instead of periodontitis-healthy individuals. In fact, chronic gingivitis patients are microbially exposed but do not present irreversible tissue destruction as chronic periodontitis patients do. Identifying the right phenotype of individuals composing the control group is essential in order to increase the study power. The statistical power of a study is strongly influenced by how the phenotype is defined and measured; misclassification or measurement errors can drastically weaken it [76,77,78]. For this reason, selecting groups based on well-defined phenotypes helps reduce heterogeneity and can improve power, which is especially important when the sample size is constrained by technical or financial limitations [77,78,79,80,81,82]. In fact, even if a traditional power calculation suggests low power, a smaller cohort with rigorously defined phenotypes and with strict inclusion and exclusion criteria may provide a more accurate estimate of the genetic risk than a much larger sample with vaguely characterized phenotypes, which only gives the illusion of strong power [76,77,80,83]. In fact, Garlet et al. [71], using this new approach, identified more SNPs significantly associated with disease risk (*IL1B*-3954, *TNFA*-308, *IL10*-592, *TLR4*-299) and yielded stronger associations and higher statistical power. They divided the founded polymorphisms into hyperresponsive variants *IL1B*-3954-T, *TNFA*-308-A, *IL6*-174-C, and hyporesponsive ones *IL10*-592-C and *TLR4*-299-G; the first ones were higher in periodontitis, and the second ones were more frequent in gingivitis, suggesting risk and protective roles, respectively. The authors suggested that phenotype definition and proper control selection are crucial in avoiding false negatives, improving study power, and clarifying genetic contributions to periodontitis.

## 8. Genetic Polymorphisms Related to Periodontitis Associated with Systemic Diseases

Periodontitis is often associated with other systemic diseases which also have inflammatory characteristics. It is important to highlight the genetic background when periodontitis co-exists with such conditions. For example, Oliveira et al. [84] explored the association between SNPs of the peptidyl arginine deaminase type 4 (*PADI4*) gene and the GTG haplotype with rheumatoid arthritis (RA), periodontitis, and neutrophil extracellular traps (NETs) in vitro, and discovered that the release of NETs by circulating neutrophils is associated with RA and periodontitis and is influenced by the presence of the GTG haplotype. Hopealaakso et al. [37] demonstrated that elevated aMMP-8 concentrations were significantly associated with both systemic conditions such as metabolic syndrome (*p* = 0.005) and diabetes (*p* = 0.03), as well as with signs of ongoing periodontal inflammation. Studies highlighted different genetic polymorphisms, demonstrating that periodontitis, for its multifactorial nature, cannot be predicted by studying just a gene or a single factor. For example, there are also ethnical differences showing that susceptibility to periodontitis varies across ethnic groups, as genotype frequencies differ among populations. This highlights the need for population-specific research to better understand genetic risk [85].

## 9. Genetic Polymorphisms Across Ethnic Groups

Genetic polymorphisms mostly associated with periodontitis are those of genes coding for *IL-1*, *IL-6*, and *TNF*-α [15,20,53,86] but, as already mentioned, the existing studies often gloss over inconsistent replication across ethnicities. In fact, it should be highlighted that polymorphisms vary across ethnic groups, and these differences should be taken into account when investigating the periodontitis genetic profile [85,87]. For example, *IL-1* has been associated with periodontitis, with differences across ethnic groups. In fact, in a Turkish population, heterozygosity for allele 1 of *IL-1α* (+4845) or homozygosity for allele 1 of *IL-1β* (+3954) increases vulnerability to localized aggressive periodontitis [88]. On the contrary, the vulnerability to localized aggressive periodontitis in Chinese populations has been associated with polymorphism at *IL-1β* (−511) [89]. Moreover, a lot of studies have demonstrated that nucleotide polymorphisms of IL-6 are correlated with the progression of periodontitis [86,90]. In particular, the presence of the *IL-6* (−174) and (−572) gene polymorphisms vary across populations. At position −174, the presence of C allele is much more frequent in Caucasian populations than in Japanese and Chinese populations. In contrast, at position −572, the presence of C allele is less frequent in Caucasian populations than in Japanese and Chinese populations. Like *IL-1*, the distribution of *IL-6* polymorphisms in African American populations stays between that of Asian and Caucasian populations [86,90,91,92,93,94,95,96].

Genetic polymorphisms linked to disease susceptibility often vary between ethnic and racial groups, particularly when comparing Asian and Caucasian populations. In fact, a genetic variant that is both common in one population and strongly associated with the disease may have less importance in another ethnic group where its frequency is low. For this reason, alleles with high prevalence in a specific population are valuable for identifying genes involved in disease risk. Nevertheless, data about the distribution of many key polymorphisms across various populations is still lacking, without sufficient replication across ethnic groups. Moreover, the relatively small effect sizes of most reported associations, combined with the lack of replication across diverse cohorts, further limits their predictive or clinical value. In order to avoid bias or misleading findings in studies associating genetic profile and periodontitis, it is crucial to enroll participants who are matched for both ethnicity and geographic background [97] and to replicate such studies in different populations.

## 10. Point-of-Care for Detecting Oral Dysbiosis

Point-of-care (POC) testing represents an innovative approach in medical diagnostics, enabling rapid, actionable results near the patient, and often eliminating the need for complex laboratory procedures and extensive training [98,99]. In periodontology, POC diagnostics are poised to revolutionize how oral diseases are detected and managed [98].

Oral dysbiosis refers to a shift in the microbial communities within the oral cavity, which is a complex environment hosting over 700 species of microorganisms. While a healthy oral microbiome maintains balance, specific pathogens, often forming supra- and subgingival biofilms, are strongly associated with the initiation and progression of periodontitis [100].

Traditionally, disease etiology focused on specific “red complex” bacteria, such as *Porphyromonas gingivalis*, *Tannerella forsythia*, and *Treponema denticola* [99], but recent insights highlight that periodontitis is linked to a global dysbiosis where health-associated species are not lost, but their proportion in the overall community decreases [101].

Detecting the disease in its early stages is crucial for timely intervention and improved patient outcomes [98].

### Current Point-of-Care Technologies for Dysbiosis Detection

Active-matrix metalloproteinase-8 (aMMP-8) is a proteolytic enzyme implicated in the degradation of connective tissue during periodontal inflammation. Elevated levels of aMMP-8 serve as a reliable indicator of ongoing tissue destruction [102,103], preceding visible clinical and radiographic signs of periodontitis [104].

Testing for aMMP-8 POC is non-invasive, typically using mouthrinse or saliva samples, and provides results rapidly within 5–10 min using a chairside lateral-flow immunotest and a digital reader (PerioSafe/ORALyzer) [102].

This allows for immediate impact on treatment planning [104]. These tests have been clinically validated globally, demonstrating utility in screening susceptible sites and patients, differentiating active from inactive periodontitis and peri-implantitis, predicting future disease progression, and monitoring treatment outcomes [105]. An aMMP-8 level over 20 ng/mL is generally considered elevated [104]. Notably, aMMP-8 POC tests have shown high specificity (96%) and good sensitivity (76–90%) for diagnosing periodontitis, with a cut-off of 20 ng/mL rarely producing false positives [105]. A study has shown that aMMP-8 levels increase with disease severity, with the highest levels detected in Stage IV periodontitis, and can differentiate periodontitis from gingivitis and healthy conditions [106].

The rapid availability of the test score allows for immediate impact on treatment planning [104], and it can also be performed by non-dental healthcare personnel or even patients themselves [105].

Regarding microbiological POC tests, technologies like quantitative polymerase chain reaction (qPCR) have replaced traditional culture-based approaches, offering higher specificity and sensitivity. Among these tests, PerioSafe^®^, iai PadoTest (ParoX GmbH, Leipzig, Germany), MyPerioPath^®^ (OralDNA^®^ Labs, Eden Prairie, MN, USA), PerioPOC^®^ (Genspeed Biotech, Linz, Austria), HR5^TM^ (MN Centre for Dental Care, Elk River, MN, USA), and OralDisk (OralDNA^®^ Labs, Eden Prairie, MN, USA) are available.

The quantitative, non-invasive, and economic PerioSafe^®^ has already been validated to function sufficiently and fluently with a single biomarker, i.e., aMMP-8 [107]. It has shown good sensitivity for active inflammation, though with methodological heterogeneity, and further research is needed to increase its diagnostic and prognostic power through combining with other possible biomarkers and integrating this test kit into periodontal risk assessment [108]. It is a commercially available FDA (USA)- and EU-approved technology [109,110,111], while iai PadoTest and MyPerioPath^®^ detect periodontal pathogens by PCR, useful as adjunctive information but with limited individual predictive value and offered as laboratory (CLIA or CE) tests without U.S. regulatory clearance [112,113]. Additionally, PerioPOC^®^ provides rapid bacterial “high-risk” panels, but clinical evidence is still inconsistent [114]. A structured comparison of the most widely used point-of-care platforms is provided in Table 4 to support clinicians in understanding their analytical targets, regulatory status, and clinical limitations.

**Figure 1:**
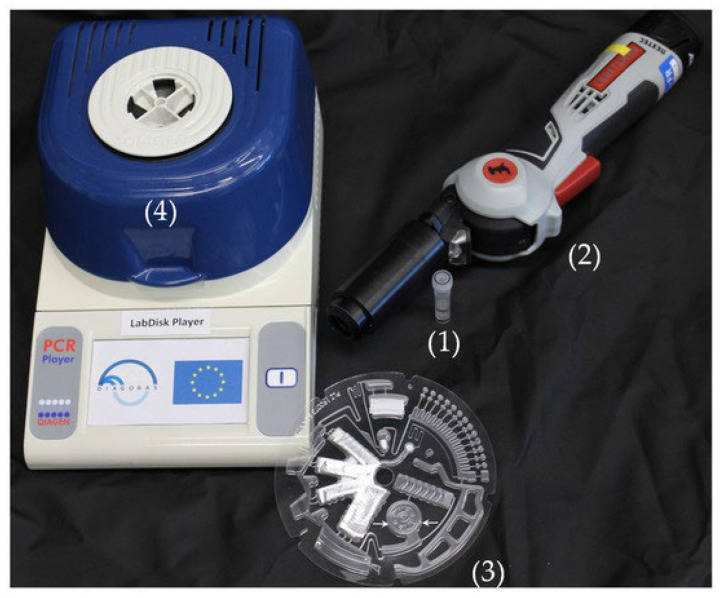
From [102], reused under CC-BY 4.0. (1) Tube contains the combination of the saliva sample, *S. marinus* control bacterium and steel beads. (2) Hand-held device (Terralyzer), where the tube is placed to carry out the mechanical lysis and homogenization of saliva. (3) The OralDisk, into which the lysate is pipetted in the chamber marked by the two white arrows. (4) The LabDisk Player instrument, which processes the OralDisk and performs real-time PCR. This system aims to integrate laboratory functions with a small chip, offering advantages like low sample volume, strong integration, and rapid response, as OralDisk delivers results in less than 3 h [115,116].

The disk-shaped microfluidic platform OralDisk (Figure 1) can detect and quantify multiple periodontitis-associated bacteria (*Aggregatibacter actinomycetemcomitans*, *Campylobacter rectus*, *Fusobacterium nucleatum*, *Prevotella intermedia*, *Porphyromonas gingivalis*, *Tannerella forsythia*, *Treponema denticola*) and caries-associated bacteria (*oral Lactobacilli*, *Streptococcus mutans*, *Streptococcus sobrinus*) from non-invasively collected whole saliva [115].

This contrasts with older laboratory-based methods like iai PadoTest, MyPerioPath, and HR5TM, which require samples to be shipped to a centralized lab, delaying results [115].

While these microbiological tests provide valuable information on bacterial types and counts, the current guidelines for antibiotic prescriptions in periodontology are primarily based on clinical assessment of disease severity, not specific pathogen detection. However, accurate microbial diagnosis can be useful for selecting patients who might benefit from antibiotic therapy, guiding antibiotic choice, and minimizing overuse, especially for eradicating bacteria with exogenous pathogen characteristics [100].

## 11. Point-of-Care and Genetic Testing for Early Detection of Gingivitis and Periodontitis

Periodontitis is a complex, multifactorially inherited disease with its phenotypic expression influenced by the interaction between genetic predisposition and environmental factors [100]. Approximately one-third of the risk for periodontitis in the population is attributed to host genetic factors [117].

Early genetic tests focused on identifying polymorphisms in genes like IL-1, which were thought to be associated with periodontitis risk. However, subsequent studies have largely failed to confirm the generalizability and practical utility of these associations, resulting in unclear benefits and limited diagnostic value [117]. Currently, no single genetic test can reliably distinguish between different forms of periodontal disease and health [38].

Genetic testing shows promise for rare, severe, single-gene, early-onset forms of periodontitis, such as those linked to mutations in the CTSC gene (cathepsin C), causing conditions like Papillon–Lefevre syndrome. In such cases, genetic information can be highly impactful, though specific data on the diagnostic utility in these scenarios are still emerging [38,117]. In fact, genetic confirmation of conditions like Papillon–Lefèvre syndrome not only supports accurate diagnosis but also has important clinical implications, including guiding family counseling and identifying potential associations with systemic diseases, enhancing patient management and risk assessment.

Despite the ability to perform whole-genome sequencing at relatively low costs, genetic profiling for common periodontitis is not routinely used in clinical practice. This is primarily due to the high cost, questionable diagnostic value compared to traditional clinical examination, and the lack of established protocols for follow-up or personalized treatment based on genetic findings [100]. Moreover, genetic tests often indicate susceptibility rather than active disease or progression [38].

### 11.1. Barriers to Clinical Adoption of Genetic Tests

Despite substantial research into genetic susceptibility for periodontitis, multiple interrelated barriers currently prevent routine clinical implementation. First, limited predictive performance constrains clinical utility: most single-gene markers show small effect sizes and PRS developed to date have modest discriminative power in general populations, reducing their ability to change management decisions beyond established clinical risk factors [118,119]. Population heterogeneity and ancestry bias undermine generalizability. The majority of GWAS and PRS derivations have been performed in European ancestry cohorts, and transferability to other ancestries is often poor; this raises concerns about equity and accuracy when applying current genetic models to diverse patient populations [117,119].

Cost-effectiveness analyses of PRS-guided interventions in other fields show mixed and context-dependent results, and robust economic evidence specific to periodontal genetic testing is lacking; payers are often reluctant to reimburse tests without demonstrated health-system benefits [120].

### 11.2. Ancestry Bias, Equity, and Ongoing Efforts to Diversify Genomic Datasets

A major limitation of current genetic studies and PRS is the strong overrepresentation of individuals of European ancestry, which reduces the generalizability and accuracy of genetic findings for other populations. Recent surveys estimate that a very large proportion of GWAS participants derive from European-ancestry cohorts, with non-European groups substantially underrepresented; this imbalance leads to reduced transferability of PRS and may worsen existing health inequities if not addressed [121,122]. The clinical implications for periodontology are direct: PRS and single-variant associations developed in European datasets typically show degraded predictive performances when applied to African, South Asian, Latino/Hispanic, and other ancestries. This means that models developed without ancestry-diverse training sets can under- or over-estimate risk in non-European patients, limiting clinical utility and risking inequitable care [123].

Encouragingly, several large international efforts are explicitly addressing dataset diversity and infrastructure for multi-ethnic genomics. Examples include the NHLBI TOPMed program (large whole-genome sequencing with diverse ancestries), the PAGE consortium (Population Architecture using Genomics and Epidemiology; focus on underrepresented groups), the All of Us Research Program (intentional recruitment of diverse US populations), African consortia such as H3Africa and recent public–private projects to build large African-ancestry reference resources, plus ongoing expansions in aggregated resources such as gnomAD which increasingly report ancestry-specific allele frequencies. These initiatives provide both reference data and cohorts needed to develop and validate ancestry-aware PRS and to discover population-specific risk loci [124].

Nonetheless, diversifying genomics raises practical and ethical challenges, community engagement, data governance, benefit sharing, and protection from misuse must be a central element of research design. Roadmaps and best-practice frameworks (including GA4GH guidance and recent academic roadmaps) stress equitable partnerships, local capacity building, and transparent access policies as prerequisites for responsible inclusion. Addressing ancestry bias requires not only expanded sampling, but also investment in local genomics capacity, phenotype harmonization across settings, and prospective validation studies that quantify predictive performance and clinical utility in each target population [123].

## 12. Future Perspectives

The future of genetic testing in periodontitis holds significant promise for transforming current diagnostic and therapeutic paradigms, moving towards a more personalized and precision medicine approach [38]. This evolution will involve the integration of genomic insights with other advanced diagnostic tools, enabling earlier detection, more accurate risk assessment, and tailored treatment strategies [117].

### 12.1. Evolution Towards Precision Periodontics

The ideal future diagnosis of periodontitis is envisioned as a comprehensive process combining traditional clinical and radiological examinations with sophisticated laboratory analyses. This shift aims to go beyond the identification of past tissue destruction by enabling the prediction of disease initiation, current activity, and future progression [100,117]. Genomic information could estimate an individual’s predisposition to disease onset, severity, and treatment response [38]. This patient-centered model underlies precision periodontics and promotes a more accurate, individualized approach compared to conventional protocols [117].

### 12.2. Polygenic Risk Scores

A key innovation in future diagnostics will be the development and validation of polygenic risk scores (PRS) [38]. These scores combine information from multiple genetic loci identified in GWAS to predict individual susceptibility to periodontitis. However, their clinical utility depends on further validation across diverse populations, as well as integration with environmental and behavioral risk factors. A generalized and clinically applicable PRS for chronic periodontitis is still under development, and future implementation will require robust evidence on its predictive value and cost-effectiveness [117].

### 12.3. Targeting Rare and Early-Onset Forms

Genetic testing already plays a clinically relevant role in rare, severe, or early-onset forms of periodontitis, such as those involving CTSC gene mutations. In these cases, genetic data can guide disease classification and support early intervention strategies [38]. In conclusion, while current genetic testing technologies offer promising tools for risk assessment and early diagnosis of periodontitis, several key steps are necessary to translate these innovations into routine practice. Future research should prioritize the development of standardized phenotype definitions, validation of polygenic risk scores across diverse populations, and the establishment of multi-ethnic longitudinal cohorts. In parallel, robust health economic evaluations and ethical frameworks are essential to ensure cost-effectiveness, clinical utility, and data governance in the application of genetic testing in dentistry.

### 12.4. Integration with Advanced Diagnostics

Future approaches will increasingly leverage multi-omics technologies—including genomics, transcriptomics, proteomics, and metabolomics [125]. Saliva and gingival crevicular fluid (GCF) are accessible biofluids suitable for such analyses [98]. Key advancements include aMMP-8 point-of-care tests and microbial profiling, as well as emerging tools such as small extracellular vesicles (sEVs), non-coding RNAs (ncRNAs), and artificial intelligence (AI). sEVs carry proteins, miRNAs, mRNAs, and DNA methylation patterns, and are under investigation as biomarkers of periodontitis [126]. Likewise, circRNAs and other ncRNAs may serve both diagnostic and therapeutic functions [127]. AI can facilitate the interpretation of large and complex “omics” datasets, translating them into actionable insights for clinical use [106].

### 12.5. Genetics, Oral Microbiome, and Protein Biomarkers in Multi-Omic Risk Models

**Figure 2:**
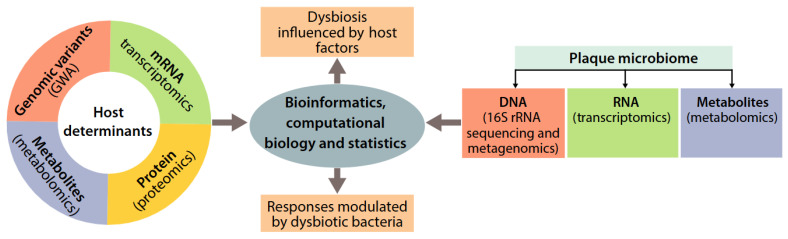
Global profile or “-omics” datasets collected from both the host and dental plaque are analyzed using advanced bioinformatics, computational biology, and statistical methods to assess how host factors influence bacterial colonization in plaque and how the host responds to an imbalanced, dysbiotic microbial community in the subgingival environment. Abbreviations: GWA, genome-wide association analysis; rRNA, ribosomal RNA. From [128] under the terms of the Creative Commons CC BY license.

Combining genetic predisposition, microbial profiles, and protein biomarkers within multi-omic frameworks allows for a deeper understanding of disease mechanisms. PRS can estimate individual risk [128], while microbiome analysis reveals functional interactions between pathogens and the host [128]. Protein biomarkers provide additional information on active disease processes and, when integrated, help identify risk patterns. Computational models, including machine learning and network analysis, can manage and interpret these complex data sources, supporting personalized prevention and treatment strategies [128,129] (Figure 2).

### 12.6. Current Limitations

To fully realize the benefits of genetic testing, several challenges must be addressed. Current tests often lack sufficient diagnostic value for common periodontitis to justify routine use [100]. Moreover, there is a need for standardized follow-up protocols and further studies evaluating clinical and economic impact [117]. Future research should include large, rigorously designed cohorts with validated diagnostic criteria [38,106].

The clinical adoption of these tools depends on demonstrating their added value and integrating them seamlessly into existing workflows [100]. Ethical considerations—such as data privacy, potential discrimination, and informed consent—also remain critical. Effective data governance frameworks and protection measures must be in place prior to widespread implementation [130,131].

## 13. Conclusions

In the literature, some studies have analyzed the genetic architecture of periodontitis, identifying some genetic polymorphisms related to its onset and progression; others have revealed the polygenic nature of the onset of periodontitis. Most polymorphisms intersect in genes that code for pro-inflammatory cytokines, highlighting the central role of inflammation in periodontitis. Promising diagnostic methods are represented by polygenic risk score and machine learning, but there are still limitations in their application. Further investigations are needed to improve genetic diagnostic methods of periodontitis and introduce them in clinical practice. Future directions should consist of conducting studies which take into account the variations in genetic polymorphisms across ethnic groups, identifying more genes related to periodontitis through WES, and overcoming current limitations of the applications of PRS and machine learning approaches.

## Figures and Tables

**Table 1:** Summary of the key cytokines involved in periodontitis.

Gene	Encoded Protein	Functional Role	Periodontitis-Associated Effects
*IL1A*	Interleukin-1 alpha	Early pro-inflammatory signaling, epithelial alarmin	Promotes neutrophil infiltration, bone resorption [23]
*IL1B*	Interleukin-1 beta	Pro-inflammatory cytokine, fever induction	Drives local inflammation and tissue degradation [24]
*IL6*	Interleukin-6	Acute-phase response, B-cell differentiation	Correlates with clinical attachment loss [25]
*TNF-α*	Tumor necrosis factor-alpha	Master regulator of inflammation, apoptosis inducer	Enhances RANKL expression and osteoclastogenesis [26]
*IL10*	Interleukin-10	Anti-inflammatory cytokine	Downregulates IL1 and TNFA, low expression linked to risk [27]
*IL17A/F*	Interleukin-17A/F	Th17 cytokines, neutrophil recruitment	Induce MMPs and RANKL, contribute to chronic inflammation [28]
*IL8* (CXCL8)	Interleukin-8	Neutrophil chemoattractant	Elevated in GCF of periodontitis patients [29]
*RANKL*	Receptor activator of NF-κB ligand	Osteoclast differentiation and activation	Increased expression causes alveolar bone loss [30]
*OPG*	Osteoprotegerin	RANKL decoy receptor, inhibits bone resorption	Decreased expression associated with bone destruction [31]
*MMP1/8/9*	Matrix metalloproteinases	ECM degradation enzymes	Collagen breakdown in periodontal ligament and alveolar bone [32]
*TLR2/4*	Toll-like receptors 2 and 4	Innate immunity, bacterial LPS recognition	Polymorphisms affect pathogen response and inflammation [33]

**Table 2:** Main genetic loci involved in periodontitis.

Gene	Encoded Protein/Feature	Putative Function	Association Summary
*SIGLEC5*	Sialic acid-binding Ig-like lectin 5	Immune inhibitory receptor on neutrophils	Genome-wide association in AgP and CP [34]
*DEFA1A3*	α-Defensins 1–3 (HNP1–3)	Antimicrobial peptides in neutrophils	CNV associated with CP risk [36]
*GLT6D1*	Glycosyltransferase-like domain protein	Potential epithelial/neutrophil glycosylation	GWAS-confirmed in CP and AgP [37]
*EFCAB4B*	EF-hand calcium-binding domain-containing 4B	Calcium signaling regulation in immune/epithelial cells	Novel locus in isolated Italian population [40]
*LAMA2*	Laminin subunit α2	ECM structure and epithelial adhesion	GWAS [41]
*ARHGAP18*	Rho GTPase-activating protein 18	Cytoskeletal remodeling and epithelial integrity	Candidate locus [43]
*HAS2/HAS2-AS1*	Hyaluronan synthase 2/Antisense RNA	Hyaluronic acid metabolism and matrix signaling	Functional candidate in epithelial barrier modulation [44]
*RP11*-61*G*19.1	Long non-coding RNA locus	Likely regulatory, function unknown	Genome-wide significant locus [42]
*HIST1H3L*	Histone H3-like protein	Chromatin and epigenetic regulation	Novel association in UK Biobank data [42]

**Table 3:** Genetic polymorphisms in gingivitis. In this table there is a summary of the genetic polymorphisms studied for gingivitis, including the ones which cause it and the protective ones against periodontitis.

Gene	Polymorphism	Study Design	Sample Size	Population	Association with Gingivitis	Replication Status	Reference
*VDR*	*VDR* FokI (rs2228570)	Cross-sectional	353	Brazilian children	No association	Not replicated	[57]
*VDR*	*VDR* BglI (rs739837)	Cross-sectional	353	Brazilian children	No association	Not replicated	[57]
*VDR*	*VDR* TaqI (rs731236)	Cross-sectional with longitudinal follow-up	51	Czech children	Associated. Recognized as risk factor for gingivitis.	Single study, replicated findings	[58]
*CD14*	*CD14* −260C/T	Case–control	590	Caucasian children with plaque-induced gingivitis	No general association, but CT/TT genotypes more frequent in gingivitis with *P. gingivalis*.	Single study	[59]
*IL-1*	*IL-1RN VNTR* (IL-1Ra)	Case–control	146	Caucasian children	Associated. IL-1RN*2 (A2) allele is protective	Single study, replicated elsewhere	[60]
*IL-10*	*IL10* −592C	Case–control	608	Brazilian (mixed ancestry, mostly Caucasian) adults affected by chronic gingivitis	Hyporesponsive SNP, prevalent in gingivitis and protective against periodontitis.	Observed in multiple studies	[71]
*TLR4*	*TLR4* −299G	Case–control	608	Brazilian (mixed ancestry, mostly Caucasian) adults affected by chronic gingivitis	Hyporesponsive SNP, prevalent in gingivitis and protective against periodontitis.	Observed in multiple studies	[71]
*TNF-α*	*TNF*-α −308G/A	Case–control	171	Adult Mexican Cohort	A/A genotype and allele A are protective, G/A is associated with increased HDL-C	Single study	[72]

**Table 4:** Comparative overview of commercially available point-of-care (POC) diagnostic platforms for periodontitis. The table summarizes each kit’s biomarker target, detection method, regulatory approval status, and limitations to assist clinicians in evaluating their clinical applicability.

Kit/Platform	Target/Biomarker	Method	Regulatory Status	Limitations
PerioSafe^®^/ORALyzer^®^	Active MMP-8 (aMMP-8)	Lateral-flow immunoassay with digital reader	FDA-approved, CE-marked	Single biomarker; methodological variability; further studies needed for integration with other markers
iai PadoTest	Periodontal pathogens	qPCR (external laboratory)	CE-marked, not FDA approved	No individual predictive value; supportive test
MyPerioPath^®^	Periodontal pathogens	qPCR (external laboratory)	CLIA-certified, not FDA approved	Limited predictive value; longer turnaround time
PerioPOC^®^/HR5™	High-risk bacterial panel	Rapid qPCR	CE-marked	Clinical evidence still limited
OralDisk	Periodontal and cariogenic bacteria	Microfluidics + real-time PCR	In development, not yet approved	Requires dedicated device; limited to research use

## Data Availability

The original contributions presented in this study are included in the article. Further inquiries can be directed to the corresponding author.
